# Mode-Coupling Generation Using ITO Nanodisk Arrays with Au Substrate Enabling Narrow-Band Biosensing

**DOI:** 10.3390/bios13060649

**Published:** 2023-06-14

**Authors:** Shuwen Chu, Yuzhang Liang, Mengdi Lu, Huizhen Yuan, Yi Han, Jean-Francois Masson, Wei Peng

**Affiliations:** 1 School of Physics, Dalian University of Technology, Dalian 116024, China; chuswdlut@163.com (S.C.); yzliang@dlut.edu.cn (Y.L.); mdlu@dlut.edu.cn (M.L.); hzyuan@dlut.edu.cn (H.Y.); 2 College of Physical Science and Technology, Dalian University, Dalian 116622, China; 3 Department of Anaesthesia, Second Hospital of Shanxi Medical University, Taiyuan 030001, China; 13753171979@163.com; 4 Département de Chimie and Centre Québécois sur les Matériaux Fonctionnels (CQMF), Université de Montréal, Montreal, QC H3C 3J7, Canada; jf.masson@umontreal.ca

**Keywords:** ITO nanodisk, nanostructure, coupling mode, biosensing, narrow band

## Abstract

Plasmonic metal nanostructures have promising applications in biosensing due to their ability to facilitate light–matter interaction. However, the damping of noble metal leads to a wide full width at half maximum (FWHM) spectrum which restricts sensing capabilities. Herein, we present a novel non-full-metal nanostructure sensor, namely indium tin oxide (ITO)–Au nanodisk arrays consisting of periodic arrays of ITO nanodisk arrays and a continuous gold substrate. A narrow-band spectral feature under normal incidence emerges in the visible region, corresponding to the mode-coupling of surface plasmon modes, which are excited by lattice resonance at metal interfaces with magnetic resonance mode. The FWHM of our proposed nanostructure is barely 14 nm, which is one fifth of that of full-metal nanodisk arrays, and effectively improves the sensing performance. Furthermore, the thickness variation of nanodisks hardly affects the sensing performance of this ITO-based nanostructure, ensuring excellent tolerance during preparation. We fabricate the sensor ship using template transfer and vacuum deposition techniques to achieve large-area and low-cost nanostructure preparation. The sensing performance is used to detect immunoglobulin G (IgG) protein molecules, promoting the widespread application of plasmonic nanostructures in label-free biomedical studies and point-of-care diagnostics. The introduction of dielectric materials effectively reduces FWHM, but sacrifices sensitivity. Therefore, utilizing structural configurations or introducing other materials to generate mode-coupling and hybridization is an effective way to provide local field enhancement and effective regulation.

## 1. Introduction

Surface plasmon polaritons (SPPs) possess subwavelength confinement of light and breakthrough diffraction limits, both of which have made for significantly progress in color filters [1,2], perfect absorbers [3,4,5], nanolasers [6,7], and chemical or biosensors [8,9]. The coherent oscillations of free electrons at the surface of metals are extremely sensitive to refractive index (RI) changes in the surrounding medium, which enables plasmonic sensors to detect molecular interactions in real time, without labels. Traditional plasmonic sensors rely on propagating SPPs excited by prism-coupling, and on localized surface plasmon resonance (LSPR)-excited nanoparticles and nanostructures. Compared to the excitation of prism-coupling, which requires large and accurate optical devices, metal nanostructures are a powerful platform for the development of multichannel and point-of-care testing (POCT) technology. Nevertheless, the inherent dispersion loss of metal materials in the visible region results in a wider full width at half maximum (FWHM), which immensely restricts the advancement of nanostructure biosensing [10,11,12]. In order to promote sensing performance, hybridization, the coupling of different resonant modes, and introducing compound materials can produce a narrower bandwidth and higher sensitivity [13,14,15].

To date, most plasmonic nanostructure sensors have been prepared using electron beam lithography (EBL), focused ion beam lithography (FIB), nanosphere lithography, nanoimprint lithography, and anodic aluminum oxide (AAO) template-based lithography [16,17]. The ideal nanofabrication technique will be low-cost, high-throughput, high-resolution, and will provide great flexibility for customizing the size and shape of the given nanostructure. Of these, AAO template-based lithography is undoubtedly the best choice for achieving large-scale and low-cost nanostructures [18].

Herein, we propose an ultra-narrow-band sensor featuring indium tin oxide (ITO) nanodisk arrays coupled with a metal substrate, and investigate the effect of various design parameters on sensing performance, using the finite difference time domain (FDTD) method. The interaction between ITO nanodisks and Au substrates leads to the excitation of the lattice mode, SPP mode and magnetic resonance mode, resulting in a bandwidth as narrow as 14 nm. This value is one fifth of that obtained by gold nanodisk arrays within the same period. In addition, changing the height of disks hardly affects the sensing figure of merit (FOM), which improves the process tolerance of the preparation. The large-scale nanostructures are fabricated through template transfer and magnetron sputtering coating. The potential of our proposed senor has been demonstrated through molecular-specific recognition.

## 2. Materials and Methods

### 2.1. Materials

In this study, 1-(3-Dimethylamino-propyl)-3-ethylcarbodiimide hydro-chloride (EDC), 11-mercaptoundecanic acid (MUA), N-Hydroxysuccinimide (NHS), and Poly dimethyl diallyl ammonium chloridewere (PDDA) were acquired from Sigma-Aldrich (Shanghai) Trading Co., Ltd., Shanghai, China. Phosphate-buffered saline (PBS, pH 7.4), bovine serum albumin (BSA), human immunoglobulin G (human IgG, Order NO. D110501), goat antihuman immunoglobulin and G (H + L) (antihuman IgG, order D111025) were purchased from Sangon Biotech (Shanghai) Co., Ltd., Shanghai, China. All biological samples were distributed in PBS solution, while other samples were used with ultrapure water (18 MΩ∙cm).

### 2.2. Preparation of Plasmonic Device Based on a Nanodisk Array Deposited on Au Substrate

The ITO–Au nanodisk arrays and full-metal nanodisk array-based plasmonic nanostructures were fabricated using template transfer and vacuum-coating. Anodic oxidation was used to fabricate an aluminum oxide (AAO) membrane with a highly ordered hexagonal array of nanoholes. Additionally, the AAO membrane has a lattice constant of approximately 450 nm, a thickness of 350 nm, and a nanohole diameter randomly varying from 260 nm to 360 nm, as reported in our previous work [19]. Then, a 100 nm-thick gold layer was deposited on the surface of the silicon wafer using magnetron sputtering. Subsequently, the AAO template was placed on surface of the gold substrate and immersed in acetone solution, resulting in the PMMA detachment of the template. This step ultimately achieved the arrangement of nanopore arrays on the substrate. Afterwards, 50 nm-thick gold layer (or 70 nm-thick ITO layer) was deposited on the surface of the template to construct Au nanodisk array (or ITO nanodisk array). Finally, the membrane was removed using epoxy tape to form the plasmonic nanostructure based on the nanodisk array deposited on the Au substrate.

### 2.3. Optical Settings

The optical response of sensor chips was acquired using a self-built reflection spectra-measuring system. A broad-spectrum light source (hL-2000, Ocean Optics Inc., Orlando, FL, USA) was used as the light source. Additionally, reflective spectra were measured using a visible/near-infrared optical fiber spectrometer (avaspec-mini4096cl, Beijing Avantes Technology Co., Ltd., Beijing, China). We adopted a 2 × 1 optic fiber coupler to connect the optical components, where the bifurcated ends are connected to a light source and spectrometer, and the combined end is connected to an optical fiber collimator. The sensor ship was placed in a polydimethylsiloxane (PDMS) flow cell, and inset and outset pipes were, respectively, joined to peristaltic pump and waste storage tank to inject and eject the sample. In addition, real-time data acquisition was processed using the customized LabVIEW program.

### 2.4. Software

This study was performed using finite difference time–domain (FDTD) algorithms to facilitate optic sensing properties. The period of the plasmonic nanodisk array was set as 450 nm, and the diameters of nanodisks are 260 nm. The thickness of the gold substrate was fixed at 100 nm, which provided the function of a mirror. The TM polarization light was incident along the *z*-direction. The periodic boundary conditions were employed along the *x* and *y* directions, and perfectly matched layer conditions were used in the *z* direction. Additionally, the size of mesh grid was set to 2 nm × 3.46 nm × 2 nm.

## 3. Results and Discussion

**Figure 1:**
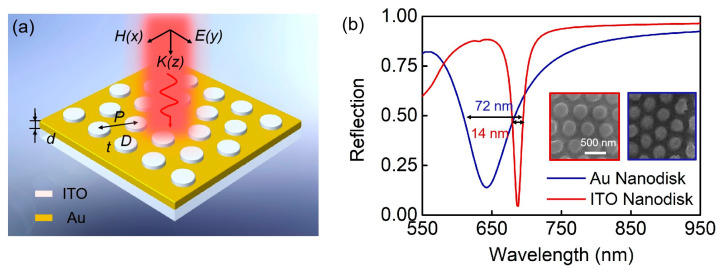
Ultra-narrow-band biosensor. (**a**) Schematic diagram of ITO–Au nanodisk arrays. (**b**) Simulated reflection spectra and SEM images of the two nanostructures. The red line and square diagrams represent ITO–Au nanodisk arrays, and the blue ones indicate full-metal nanodisk arrays.

**Figure 2:**
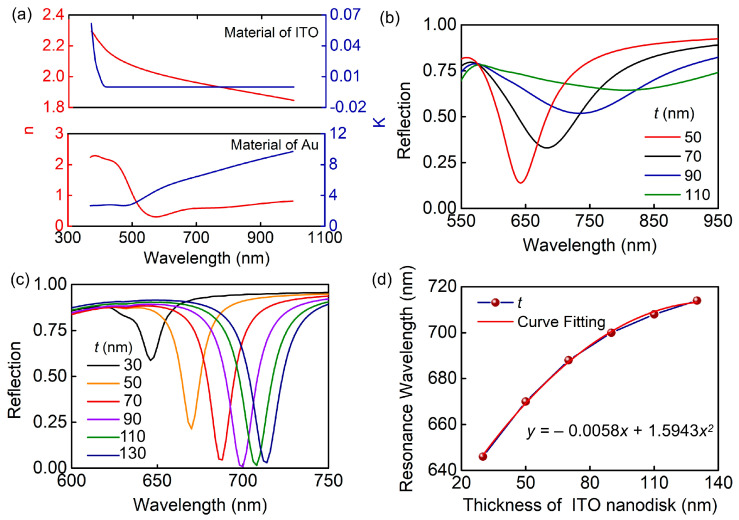
Optical properties of ITO–Au nanodisk arrays and full-metal nanodisk arrays under normal incidence. (**a**) The refractive index (*n*) and extinction coefficient (*k*) of the material for ITO and Au using ellipsometry. (**b**) Spectral shift as the thickness of Au nanodisks (*t*). (**c**) Spectral shift and (**d**) resonance wavelength as the thickness of ITO nanodisks (*t*).

Figure 1a illustrates the schematic and geometric parameters of our proposed ultra-narrow-band biosensor. ITO nanodisks arranged in hexagonal arrays are integrated into the gold substrates, producing ITO–Au nanodisk arrays. The period of the unit cell is *P*. The diameter and thickness of the nanodisk are *D* and *t*, respectively. The permittivity of Au and ITO in the visible and near-infrared regions are obtained from experimental results. We obtained the refractive index (*n*) and extinction coefficient (*k*) of the material using ellipsometry, as shown in Figure 2a. The refractive index of the silica substrate is 1.46.

In order to clarify the spectral characteristics of the proposed structure, we have compared it with Au nanodisk arrays on the Au substrate, assuming full-metal nanostructures. Figure 1b depicts the calculated spectra of reflection for the patterned Au nanodisk and the ITO nanodisk integrated with a Au layer when the refractive index of the cover layer is *n* = 1.3313. Our proposed ITO–Au nanodisk arrays reduce the FWHM down to 14 nm, leading to an ultra-narrow-band resonance mode. Significantly, the FWHM of the full metal nanodisk arrays is five times that of the aforementioned ITO–Au nanodisk arrays. In order to reveal the optical properties and sensing applications of the proposed structure, we conducted a comparative analysis of the ITO–Au nanodisk arrays and full-metal nanodisk arrays. The insets show the experimentally obtained structure with a highly ordered hexagonal array of nanodisks.

Figure 2 illustrates the simulation results for both of the nanodisk arrays. When the other structural parameters are fixed, the thickness (*t*) of the nanodisks has different effects on the reflection spectra of the two structures. As shown in Figure 2b,c, the resonance wavelength shifts to the red side for both structures with an increase in thickness. Nevertheless, the reflection depth decreases sharply, accompanied by a significant broadening of FWHM for the full metal nanodisk arrays. In contrast, the reflection depth of the ITO–Au nanodisk arrays gradually increases. When it reaches 70 nm, the resonance intensity hardly changes. In this condition, the wavelength dependency of the thickness of the nanodisk can be disregarded. In addition, the relationship between thickness and resonance wavelength is established using polynomial fitting, as shown in Figure 2d. The resonance wavelength position can be flexibly regulated by controlling the thickness of the nanodisk.

**Figure 3:**
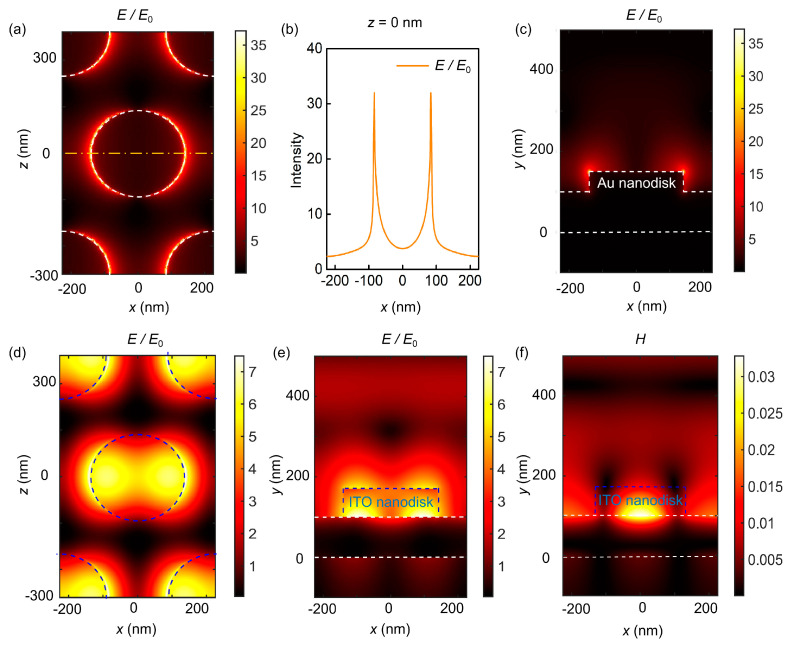
Electromagnetic field distribution of resonance wavelengths. For the full metal nanodisk arrays, electric field distributions (**a**) in the *xz* plane for *y* = 150 nm, (**b**) the variation along *z* direction of orange dotted line, and (**c**) in the *xy* plane. For the ITO–Au nanodisk array structure: electric field distributions in (**d**) *xz* plane for *y* = 170 nm, (**e**) the *xy* plane, and (**f**) magnetic field distribution in the *xy* plane. The white dashed line indicates metal material, while the blue dashed line indicates ITO material.

In order to understand the underlying physics of the two modes excited by the ITO–Au nanodisk arrays and the full-metal nanodisk arrays, Figure 3 shows the distribution of the electromagnetic field in the *xy* plane and *xz* plane. For the full-metal nanodisk arrays, the energy is localized at the edge of nanodisks, as shown in Figure 3a,c. Additionally, Figure 3b reveals the variation of electric field intensity along the *z* direction of the orange dotted line in Figure 3a. The opaque gold film hinders the transmission of incident light to the silica substrate, and forms coupling modes based on the film and Au nanodisk arrays at a wavelength of 648 nm. The diffraction coupling of local dipole modes leads to the excitation of the surface plasmon Bloch mode [20]. The fields are normalized to the field amplitude of the incident light (*E*/*E*_0_). The white dashed boxes indicate metal material, while blue ones indicate ITO material. For the ITO–Au nanodisk arrays, the electric field at the wavelength of 684 nm is mainly localized at the interfaces between the ITO nanodisks and the Au layer, and the surface of the measuring solution (Figure 3d,e). The near-field intensity decreases exponentially, which is consistent with the characteristics of the propagating surface plasmon mode for infinite planar noble films. The generation of the dipolar plasmon mode originates from the lattice resonance of the hexagonal ITO nanodisk diffraction field. In addition, the narrow-band excitation in this structure is also attributed to the magnetic resonance that combines the magnetic field distribution (i.e., strong magnetic field distribution in the ITO nanodisks) with the charge distribution on the upper and underneath surfaces of the disks, as shown in Figure 3f. Therefore, the narrow-band resonance is the result of the coupling of the propagating surface plasmon mode resonances on the upper and bottom surfaces of the gold film with the magnetic resonance of the nanodisk arrays. That is, changing the height of the disks can affect the resonance wavelength. The numerical results indicate that the introduction of ITO nanodisks effectively reduces the inherent loss of metal materials. Unfortunately, most of the excited electric field is hidden beneath nanodisks, which is unfavorable for biosensing.

In order to evaluate the overall performance of the proposed coupling sensors, we adopt the widely used sensitivity and bulk FOM, wherein sensitivity (*S*) can be defined as the amount of wavelength shift ∆*λ* as a function of the induced RI change [21,22]:

(1) $$S=\frac{\Delta \lambda }{\Delta n}$$

and the FOM depends on the ratio of sensitivity *S* and FWHM [22,23]:

(2) $$\mathrm{FOM}=\frac{S}{\mathrm{FWHM}}$$

It is worth mentioning that the definition of *S* here refers to bulk sensitivity.

**Figure 4:**
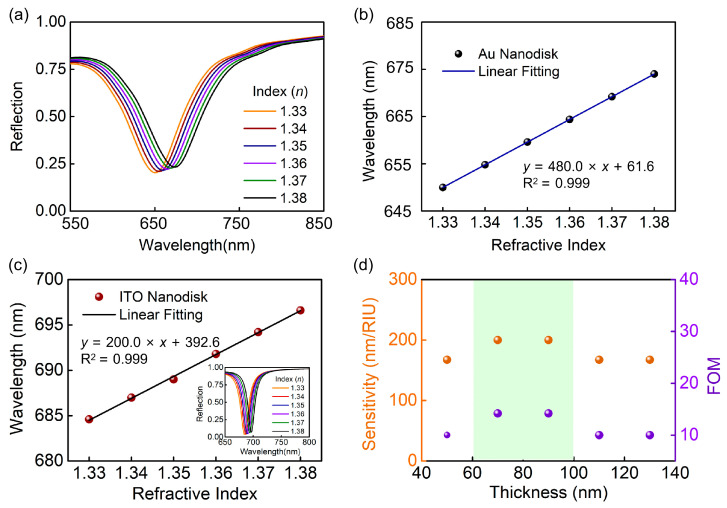
Characterization of the sensing performance of full metal nanodisk arrays and ITO–Au nanodisk arrays. (**a**) Stimulated reflection spectra with ambient refractive indexes, and (**b**) the dependence of resonance wavelengths on the ambient refractive index for full metal nanodisk arrays. (**c**) Stimulated reflection spectra with ambient refractive indexes, the dependence of resonance wavelengths on the ambient refractive index, and (**d**) the influence of different thicknesses on sensitivity and sensing FOM for the ITO–Au nanodisk arrays.

As shown in Figure 4, we have calculated the reflection of proposed structure with a cover layer for different refractive indices. For the full-metal nanodisk arrays, the position of the dip has redshift from 650 nm to 675 nm when the ambient refractive index increases from 1.33 to 1.38 (Figure 4a), while the value of the reflective dip and the line shape of the resonance remains constant. Figure 4b extracts the positions of reflection dip from Figure 4a to plot them as a function of the refractive indices, and represents the blue line as the line fitting. The slope of resonance mode is 480 nm/RIU, and the bulk FOM is 6.67. For the ITO–Au nanodisk arrays, the inset of Figure 4c demonstrates the dependence of the reflection spectra on the change in the ambient refractive indexes. Additionally, the resonance wavelength has a redshift with the increase in the ambient refractive index from 1.33 to 1.38, and the line shape of the spectra remains constant, as illustrated in Figure 4c. The slope of plasmonic resonance is 200 nm/RIU and the bulk FOM is 14.28 when the thickness is 70 nm, which is twice the sensitivity of the full metal nanodisk arrays. Furthermore, we discuss the changes in sensitivity and FOM under different thicknesses. The results show that the values remain unchanged in the range of 70–90 nm, which greatly improves the tolerance of our proposed structure to the preparation process (Figure 4d).

**Figure 5:**
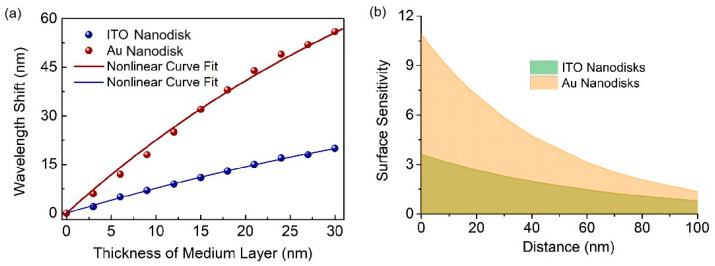
Simulation results of surface sensitivity for the two nanostructures. (**a**) Resonance wavelength shifts with varied thickness of the cover layer. (**b**) The second-order surface sensitivity curve as a function of the adsorbate thickness.

In essence, bulk sensitivity describes the measurement of accessible field regions from the surface of nanostructure sensors to infinity. Nevertheless, it is inappropriate for biological molecules, such as proteins, DNA, and viruses. In order to more accurately judge the performance of sensor response, the surface sensitivity is used for evaluation. It describes the change caused by the refractive index of the thin layer near the sensor surface. To quantify the performance of sensors, a molecular layer with a refractive index of 1.45 is covered on the surface. We investigate the spectra of reflection with varying thicknesses of molecular layer. Obviously, the resonance wavelengths of both structures undergo redshift as the thickness of the protein layer increases from 0 to 30 nm. The dependence of resonance wavelength on the distance from surface is considered, so we fit the data in Figure 5a to exponential curves. The formula is as follows [19,24]:

(3) $$\Delta \lambda =\Delta \lambda_{\infty }(1-e^{-d/l_{d}})$$

Wherein Δ*λ*_∞_ denotes the wavelength shift induced by a layer with an infinite thickness. d is the thickness of additional layer, assuming a molecular layer, and *l*_d_ is related to the decay length of the electromagnetic field. As shown in Table 1, we obtained Δ*λ*_∞_ = 119.8 nm and *l*_d_ = 48.0 nm^−1^ for the full-metal nanodisk arrays, and Δ*λ*_∞_ = 54.8 nm and *l*_d_ = 66.2 nm^−1^ for the ITO nanodisk arrays. This further verifies that both structures are appropriate for sensing applications.

The surface sensitivity is defined as the second derivative of the index and thickness of the adsorbate [25,26]:

(4) $$S_{surf}=\frac{\partial^{2}\lambda }{\partial d\partial \Delta n}=\frac{\partial \Delta \lambda_{\infty }}{\partial \Delta n}\frac{\partial (1-e^{-d/l_{d}})}{\partial d}=\frac{\Delta \lambda_{\infty }}{\Delta nl_{d}}e^{-d/l_{d}}$$

where ∆*n* = *n*_alum_ − *n*_water_ = 1.56 − 1.33 = 0.23 is the refractive index change. Figure 5b shows the dependence of surface sensitivity for both resonance modes of the two nanostructures on the distance from the structure surface. For the ITO–Au nanostructure, partial near-field enhancement is leaked onto the surface of the metal substrate, which results in a lower sensing performance and surface sensitivity compared to the full-metal nanostructure.

**Figure 6:**
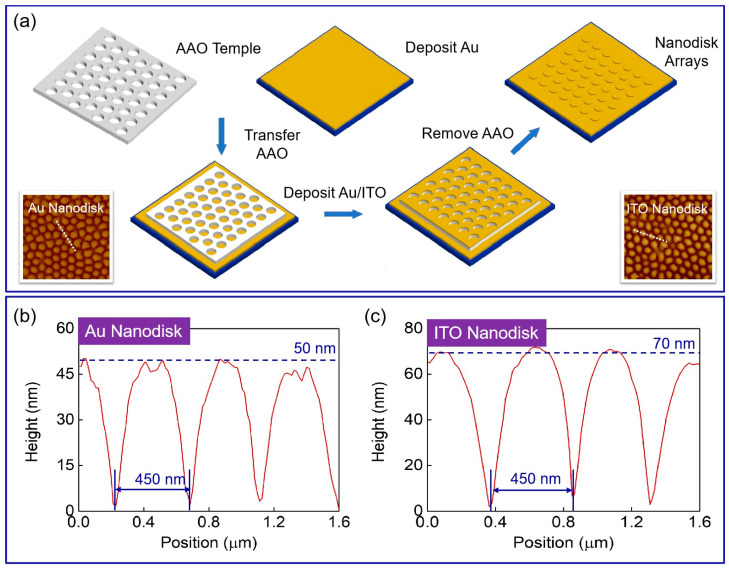
The fabrication of nanodisk-based structures and AFM images. (**a**) Scheme for producing low-cost nanodisk structures using template transfer. Insets show AFM images of full-metal nanodisk arrays and ITO–Au nanodisk arrays. (**b**) White line profiles across the patterns for full-metal arrays. The period is 450 nm and the height of the Au disks is 50 nm. (**c**) White line profiles across the patterns for ITO–Au nanodisk arrays. The period is 450 nm and the height of the ITO disks is 70 nm.

To experimentally investigate the sensitivity of our proposed nanostructure arrays on Au substrate, we utilized a template transfer and magnetron sputtering fabrication scheme, as illustrated in Figure 6. The AAO template has good periodicity, which is consistent throughout the preparation of full-metal nanodisk arrays, with the only difference being the second deposition of metal film. White line profiles across the patterns are shown in Figure 6b,c; these indicate the thickness of the nanodisks in the two prepared structures (i.e., 50 nm for gold nanodisks and 70 nm for ITO).

**Figure 7:**
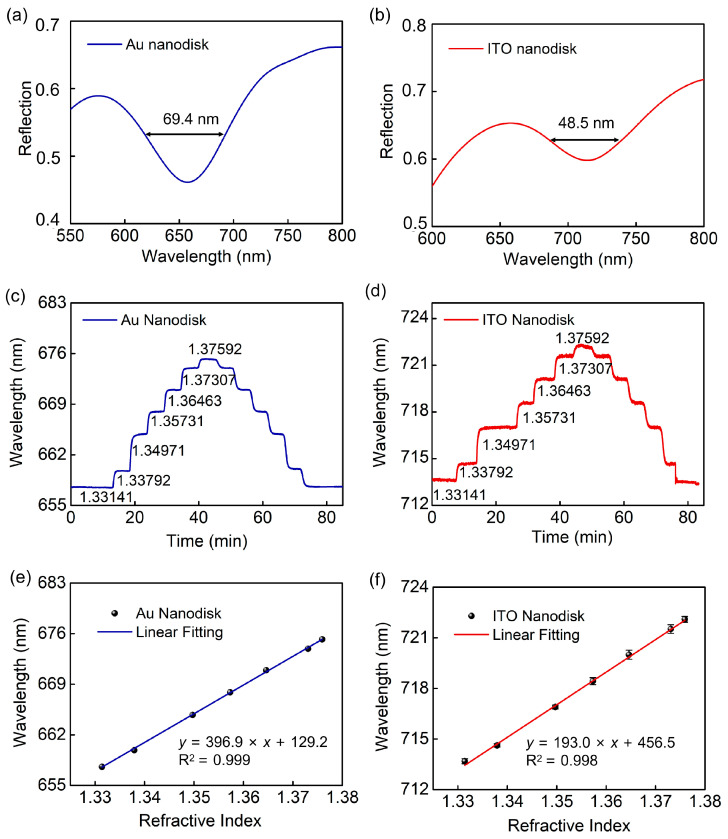
Measured dependence of resonance wavelength with refractive indices of different salt solution analytes. For full-metal nanodisk arrays: (**a**) a typical spectrum in water, (**c**) temporal dependence, and (**e**) linear fitting of spectral positions. For ITO–Au nanodisk arrays: (**b**) typical spectrum in water, (**d**) temporal dependence, and (**f**) linear fitting of spectral positions.

We used a home-built reflective spectral detection system, including a light source, spectrometer and the LabVIEW program, to achieve sensing detection and data acquisition. Figure 7 shows the reflection spectra and wavelength response of two structures under normal incidence. Figure 7a,b reveal that the resonance wavelength and FWHM are 660 nm with 69.4 nm for full-metal nanodisk array, and 712 nm with 48.5 nm for ITO–Au nanodisk array, respectively. However, the bandwidth is notably wider compared to the simulation results, due to the experiment’s template quality. The sensitivity was evaluated by changing the refractive index of the measured sodium chloride solution, which was measured using an Abbe refractometer. The prepared structures have been immersed in different concentrations of refractive indices ranging from 1.3314 to 1.3759. As the refractive index increases, the position of the resonance wavelength experiences redshift, as displayed in Figure 7c,d. The relationship between the refractive index and resonant wavelength assumes linear fitting, as demonstrated in Figure 7e,f. Additionally, the sensitivity of the full-metal nanodisk arrays is *S*_Au_ = 396.0 nm/RIU, while that of the ITO–Au nanodisk arrays is *S*_ITO_ = 193.0 nm/RIU.

**Figure 8:**
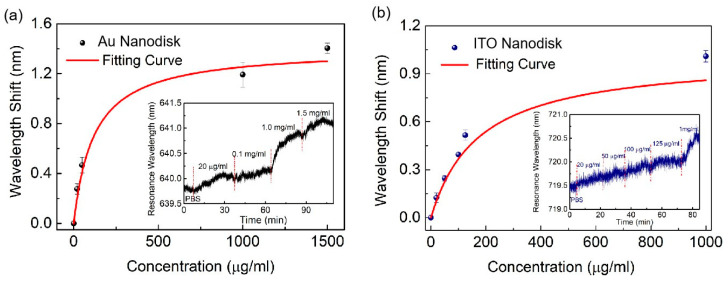
Probing of IgG with resonance modes for the two nanostructures: (**a**) full-metal nanodisk arrays; (**b**) ITO–Au nanodisk arrays.

To investigate the potential of the developed nanostructure for biosensing applications, we utilized plasmonic resonances for analyzing molecular binding events between IgG and anti-IgG. The specific surface modification process is categorized two ways. One pertains to the full-metal nanostructure array [27,28]. Initially, the sensor chip is immersed in an MUA of 10 mM for 12 h to self-assemble the carboxylic layer, which is then dried with nitrogen. Later, an EDC/NHS mixed solution (0.5 M and 0.55 M) is used to activate the hydroxyl groups on the nanostructure surface for 20 min at 4 °C, and the surface is cleaned with deionized water. Subsequently, 0.1 mg/mL anti-IgG phosphoric acid buffer solution (PBS, PH = 7) is added to the surface for 30 min, followed by washing with PBS buffer. Lastly, the surface is coated with 1 mg/mL of BSA solution for 15 min to prevent unmodified anti-IgG. Subsequently, surface functionalization is completed. Human immunoglobulin IgG samples of 0.02, 0.1, 1.0, and 1.5 mg/mL are injected, and with an increase in concentration, the resonance wavelength has redshift. Through calculation and fitting, it is deduced that the equilibrium dissociation constant and the maximum wavelength shift are 120 μg/mL and 1.40 nm, respectively (Figure 8a).

The aforementioned method is applicable to thiol modification interactions with gold material, but not to ITO material. Therefore, in order to detect IgG proteins for the ITO–Au nanodisk arrays, we utilized a positive and negative charge adsorption. The IgG displays a positive charge with a PH < 6.9, and a negative charge with a PH > 6.9 [29,30]. In addition, PDDA was utilized as a strong cationic polyelectrolyte layer. The detection process involves immersing the sensor chip in PDDA solution with a 1% mass fraction for 15 min, and then cleaning the surface with deionized water. We then immobilized anti-IgG with 1 mg/mL on the sensor surface using electrostatic adsorption for 30 min, followed by cleaning with PBS solution. Finally, termination processing was carried out using BSA solution. IgG samples with different concentrations were detected. Similarly, the resonance wavelength redshifts as the concentration increases in ITO nanostructures. Through calculation and fitting, the equilibrium dissociation constant and the maximum wavelength shift were found to be 180 μg/mL and 1.00 nm, respectively (Figure 8b). The results indicate that biosensing can be achieved through plasmonic full-metal nanodisk arrays and ITO–Au nanodisk arrays, which helps to optimize sensing performance and enriches detection systems.

## 4. Conclusions

In this paper, we developed a coupling nanostructure generated by ITO nanodisks on an Au substrate. This plasmonic nanostructure effectively decreases the bandwidth and improves the sensing FOM in comparison with full-metal nanostructures. The interaction between the ITO nanodisks and the Au substrate produces both SPP mode and magnetic resonance, leading into an ultra-narrow bandwidth for coupling resonance mode. We have demonstrated the biosensing application of these ITO–Au nanodisk arrays through specific antigen–antibody binding, with a detection limit of 180 μg/mL. This scheme provides a feasible option for coupling hybrid mechanisms, alongside potential promising biosensing performance.

## Figures and Tables

**Table 1:** The comparison of surface sensitivity for the two structures.

	Δ*λ*_∞_	*l*_d_
Full-metal nanodisk arrays	119.8 nm	48.0 nm
ITO nanodisk arrays	54.8 nm	66.2 nm

## Data Availability

Not applicable.
